# Alpha Rhythms Reveal When and Where Item and Associative Memories Are Retrieved

**DOI:** 10.1523/JNEUROSCI.1982-19.2020

**Published:** 2020-03-18

**Authors:** María Carmen Martín-Buro, Maria Wimber, Richard N. Henson, Bernhard P. Staresina

**Affiliations:** ^1^Laboratory of Cognitive and Computational Neuroscience (UCM-UPM), Center for Biomedical Technology, 28223 Pozuelo de Alarcón, Madrid, Spain,; ^2^Faculty of Health Sciences, King Juan Carlos University, 28922 Alcorcón, Madrid, Spain,; ^3^School of Psychology and Centre for Human Brain Health (CHBH), University of Birmingham, B15 2TT Birmingham, United Kingdom, and; ^4^MRC Cognition and Brain Sciences Unit and Department of Psychiatry, University of Cambridge, CB2 7EF Cambridge, United Kingdom

**Keywords:** alpha, episodic memory, hippocampus, MEG, oscillations, parietal cortex

## Abstract

Memories for past experiences can range from vague recognition to full-blown recall of associated details. Electroencephalography has shown that recall signals unfold a few hundred milliseconds after simple recognition, but has only provided limited insights into the underlying brain networks. Functional magnetic resonance imaging (fMRI) has revealed a “core recollection network” (CRN) centered on posterior parietal and medial temporal lobe regions, but the temporal dynamics of these regions during retrieval remain largely unknown. Here we used Magnetoencephalography in a memory paradigm assessing correct rejection (CR) of lures, item recognition (IR) and associative recall (AR) in human participants of both sexes. We found that power decreases in the alpha frequency band (10–12 Hz) systematically track different mnemonic outcomes in both time and space: Over left posterior sensors, alpha power decreased in a stepwise fashion from 500 ms onward, first from CR to IR and then from IR to AR. When projecting alpha power into source space, the CRN known from fMRI studies emerged, including posterior parietal cortex (PPC) and hippocampus. While PPC showed a monotonic change across conditions, hippocampal effects were specific to recall. These region-specific effects were corroborated by a separate fMRI dataset. Importantly, alpha power time courses revealed a temporal dissociation between item and associative memory in hippocampus and PPC, with earlier AR effects in hippocampus. Our data thus link engagement of the CRN to the temporal dynamics of episodic memory and highlight the role of alpha rhythms in revealing when and where different types of memories are retrieved.

**SIGNIFICANCE STATEMENT** Our ability to remember ranges from the vague feeling of familiarity to vivid recollection of associated details. Scientific understanding of episodic memory thus far relied upon separate lines of research focusing on either temporal (via electroencephalography) or spatial (via functional magnetic resonance imaging) dimensions. However, both techniques have limitations that have hindered understanding of when and where memories are retrieved. Capitalizing on the enhanced temporal and spatial resolution of magnetoencephalography, we show that changes in alpha power reveal both when and where different types of memory are retrieved. Having access to the temporal and spatial characteristics of successful retrieval provided new insights into the cross-regional dynamics in the hippocampus and parietal cortex.

## Introduction

Episodic memory, our ability to remember past events and experiences, is a key part of human cognition. Intriguingly though, some memories remain faint, eliciting a sense of familiarity at best, while others are vivid and bring back a wealth of associations (Yonelinas, 2002). Investigation of the neural mechanisms supporting memory recall was ignited by electroencephalography (EEG) studies. A consistent finding in these studies is a characteristic deflection of the event-related potentials (ERPs) for old (previously encountered) versus new (not previously encountered) stimuli. This “old/new” effect is most pronounced over left posterior sensors and unfolds between 500 and 1000 ms after cue onset (Sanquist et al., 1980; for a review see Rugg and Curran, 2007; Staresina and Wimber, 2019). In parallel, functional magnetic resonance imaging (fMRI) studies have consistently shown a core brain network, featuring parietal and medial temporal regions, that is engaged during successful recollection (Hayama et al., 2012; Rugg and Vilberg, 2013). However, due to inherent limitations of both methods (relatively poor spatial resolution of scalp ERPs and poor temporal resolution of fMRI), it is unclear whether the cue-evoked ERPs reflect engagement of the “core recollection network” and whether engagement of the core recollection network observed via fMRI is temporally linked to the moment of retrieval, as opposed to prestimulus/preparatory deployment of attention or postretrieval monitoring (Levy, 2012; Sestieri et al., 2017). Moreover, it is challenging to disentangle the temporal dynamics within the recollection network with fMRI, allowing only speculation about whether parietal regions drive the hippocampus in a top-down manner during successful recall or whether the hippocampus provides a bottom-up signal to parietal regions (Wagner et al., 2005; Ciaramelli et al., 2008; Vilberg and Rugg, 2008). Direct intracranial recordings would provide the desired temporal and spatial resolution, but comprehensive coverage of both parietal and mediotemporal areas is rare, and more sophisticated retrieval paradigms (probing different types of memory) are challenging to conduct with patients (Foster et al., 2015; Gonzalez et al., 2015).

**Figure 1.**
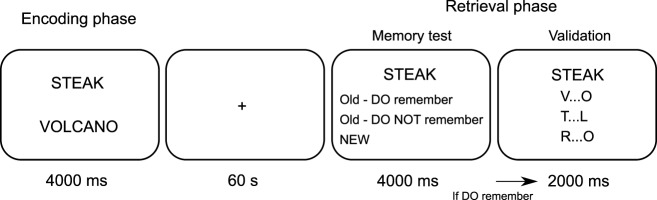
Experimental procedure. During the study phase (“encoding”), participants saw word pairs under deep or shallow processing tasks. During the subsequent test phase (“retrieval”), one word of the previously presented pairs was shown, intermixed with unstudied new words (“lures”). Participants indicated with one button press whether they thought the given word was new, the word was old but they did not remember the paired associate or the word was old and they recalled the paired associate. In the latter case, a second screen appeared to validate recall accuracy, providing three first-last letter combinations of which one corresponded to the target association. Analyses focused on correct identification of lures (CR), correct identification of old words without recalling the paired associate (IR) and correct identification of old words along with correctly recalling the paired associate (AR).

That said, one measure that may integrate the strengths of EEG and fMRI recordings are oscillatory patterns in the alpha frequency band (8–12 Hz). On the one hand, simultaneous EEG-fMRI recordings have revealed a strong link between blood-oxygenation-level-dependent (BOLD) signal increases and decreases in alpha power ('desynchronization') (Laufs et al., 2003; Moosmann et al., 2003; Meltzer et al., 2007; Scheeringa et al., 2011). On the other hand, modeling and empirical work suggests that sustained and late ERPs might reflect asymmetric amplitude fluctuations in the alpha band, such that e.g., oscillatory peaks become more pronounced than troughs over time (Mazaheri and Jensen, 2008). We thus hypothesized that alpha desynchronization not only differentiates between different types of episodic retrieval in the time domain (from ∼500 ms onward), but that this effect spatially maps onto the core recollection network, thus pinpointing its purported role in peristimulus retrieval. Capitalizing on the increased spatial resolution of magnetoencephalography (MEG) over EEG (Lopes da Silva, 2013; Baillet, 2017), we used a memory retrieval paradigm (Fig. 1) in which participants indicated whether a given word was: (1) new (correct rejection, CR), (2) old but they could not recall the paired associate (item recognition, IR), or (3) old and they also recalled the paired associate (associative recall, AR). Examination of the condition-specific time courses of alpha power showed that AR effects indeed unfolded after IR effects. Then projecting the data into source space indicated that these effects were carried by the core recollection network, including hippocampus and posterior parietal cortex. Critically, by integrating temporal and spatial signal properties, we suggest that the hippocampus provides a bottom-up signal to parietal cortex during successful recall. Thus, our results show how alpha oscillations reveal the intricate spatiotemporal dynamics of memory retrieval.

## Materials and Methods

### 

#### 

##### Participants.

Our sample consisted of 15 healthy, right-handed individuals (9 females; mean age: 24 years, range: 18–37) who gave written informed consent. All procedures were approved by University of Cambridge Psychology Research Ethics Committee.

##### Paradigm.

The experiment was conducted inside the MEG shielded room with the participant seated upright. A schematic diagram of the experimental paradigm is shown in Figure 1. Participants completed eight encoding-retrieval runs with 60 s before and after each encoding phase in which they were asked to look at a central fixation cross. During encoding, participants were presented with pairs of English nouns. To obtain experimental leverage on different memory outcomes (item and associative memory), we used a levels-of-processing manipulation during encoding (Craik and Lockhart, 1972): a 'syllable task' in which participants indicated how many of the two words contained 2 syllables (0, 1 or 2; shallow encoding), and an 'imagery task' in which participants vividly imagined the two objects interact and indicated their imagery success (low, medium, high; deep encoding). Each word pair remained on the screen for 4 s regardless of the participant's response. Incidental to the encoding task, a flickering background, flickering at 8.6 or 12 Hz, was presented on the left or right side of the screen which participants were instructed not to pay attention to. The flicker manipulation during encoding is beyond the scope of the current manuscript, but counterbalancing ensured that deep and shallow encoding trials were equally often presented with both flicker rates and at both visual hemifields. Each encoding block contained 28 word pairs, with deep and shallow tasks alternating every 7 trials. During the subsequent retrieval block, participants were presented with one randomly chosen word from each of the 28 previously seen pairs as well as 14 novel nouns. First, participants indicated if the word was: (1) new, (2) old but they could not remember the paired associate, or (3) old and they also remembered the paired associate. The response was collected with a single button press (hand assignment counterbalanced across participants) and the word remained on the screen during 4 s regardless of the participant's response. When the third option was selected, a validation screen appeared for 2 s and the participants had to choose which of three first-and-last letter combinations corresponded to the remembered paired associate. This two-step structure served as a means of accuracy assessment while holding the stimulus display and response options constant for the initial 4 s of the trial. Preceding each trial, a fixation cross was displayed during a jittered intertrial interval of 850 to 1150 ms. For subsequent analyses, the following three conditions of interest were defined: correct rejection (CR; trials in which participants correctly identified new words); item recognition (IR; trials in which participants indicated they recognized an old word but did not recall the paired associate), and associative recall (AR; trials in which participants indicated they recognized an old word and recalled the paired associate, followed by a correct response during validation). To restrict our analyses to correct memory trials, we excluded misses (trials in which old items were incorrectly identified as new), false alarms (trials in which new items were incorrectly identified as old) and trials in which participants first indicated they recalled the word plus its paired associate but then gave an incorrect response during verification. In terms of nomenclature, we define an item recognition effect as the difference between IR and CR and an associative recall effect as the difference between AR and IR. The experiment was programmed in MATLAB using the Psychophysics Toolbox extensions (Brainard, 1997; Pelli, 1997).

##### MEG recordings.

Data were recorded in a magnetically shielded room using a 306-channel VectorView MEG system (Elekta). Data were sampled at 1 kHz with a high-pass filter of 0.03 Hz. Head position inside the MEG helmet was continuously monitored by means of five head position indicator (HPI) coils. A 3D digitizer (Fastrack; Polhemus) was used to record the location of the HPI coils and the general head shape relative to three anatomical fiducials (nasion, left and right preauricular points). To track eye movements and blinks, bipolar electrodes were attached to obtain horizontal and vertical electrooculograms (HEOG and VEOG).

##### MEG preprocessing.

MEG data were cleaned of external noise using the Maxfilter 2.0 software (Elekta), applying the Signal-Space Separation (SSS) method with movement compensation (Taulu and Simola, 2006), correlation limit of 0.9 and time window of 10 s. Next, data were preprocessed and subsequently analyzed with the FieldTrip toolbox (Oostenveld et al., 2011) running in MATLAB. Data were segmented into trial epochs from −2 to 7 s time locked to stimulus onset and then downsampled to 200 Hz. After discarding trials with muscle and jump artifacts by trialwise inspection, an Independent Component Analysis was computed. Independent components reflecting eye movements and heartbeat were identified by visual inspection of component scalp topographies, time courses and comparison with EOG raw time-series. Raw data and ICA topographies of both sensor types (gradiometers and magnetometers) were visualized in parallel to ensure we discard the same components. Noise components were removed and clean trials were visually inspected again to identify and remove any remaining artifact. Across participants, an average of 15% (SD = 17%, range: 1–60%) of all trials were discarded. The CR condition contained an average of 79 trials (SD = 21, range: 30–109), the IR condition contained and average of 74 trials (SD = 33, range: 14–135) and the AR condition contained an average of 55 trials (SD = 35, range: 10–151).

**Figure 2.**
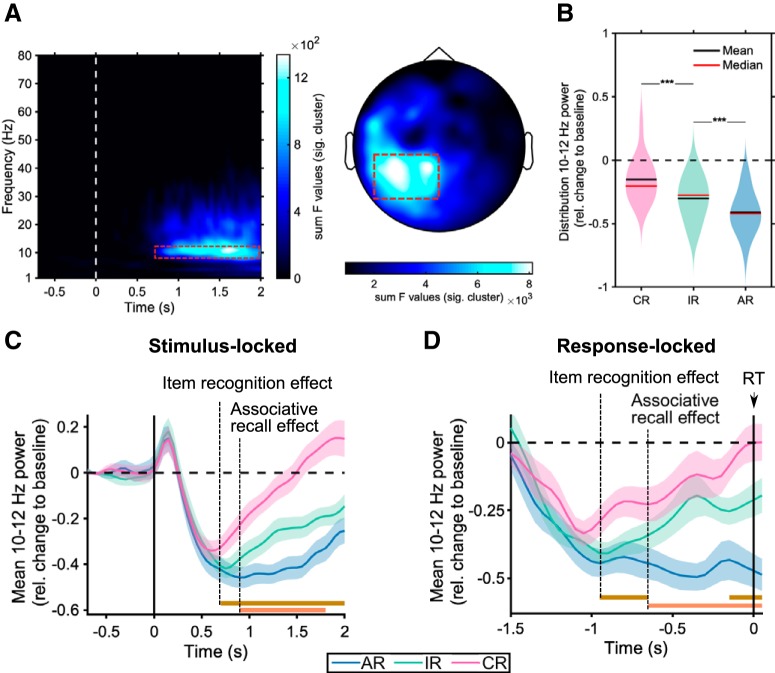
Sensor space results. ***A***, ANOVA results for the comparison of CR, IR and AR TFRs revealed a significant cluster from 0.7–2 s at left posterior sensors with a peak at 10–12 Hz. TFR plot (left) depicts the sum of F-values across all significant sensors of the cluster. Topoplot (right) shows the sum of F-values across all significant time/frequency bins of the cluster. ***B***, Distribution, mean and median of alpha power for each memory condition collapsed across left posterior sensors from 0.7–2 s in the 10–12 Hz frequency range (red dashed boxes in ***A***), showing a relative power decrease (“desynchronization') modulated by memory outcome. ***p < 0.001, paired samples *t* test. ***C***, Alpha power (10–12 Hz) time courses, collapsed across left posterior sensors (cf. Fig. 2*A*). ***C***, Stimulus-locked and (***D***) Response-locked averages across participants (±SEM). Dashed vertical lines highlight onsets at which item recognition memory effects (IR vs CR) and associative recall effects (AR vs IR) effects unfold, and brown and orange horizontal lines depict the significant clusters for the respective paired-samples *t* tests (all *p* < 0.005). For robustness of results to a wider range of sensors and frequency bands, see Figure 2-1. For analogous analyses on magnetometer data, see Figure 2-2.

The main analyses in sensor and source space were conducted using the 204 planar gradiometer data. Note that a highly similar network emerged when using magnetometer instead of gradiometer data (Fig. 2-2; see Garcés et al., 2017 for discussion on the choice of the sensor types to use in source reconstruction analyses).

##### Sensor space time–frequency analysis and statistics.

Frequency decomposition was obtained for each trial using fast Fourier transform (FFT)-based sliding window analysis, progressing in 50 ms steps. The window length was optimized for each frequency from 1 to 80 Hz, with a minimum of 200 ms and 5 cycles (for instance, using 500 ms/5 cycles for 10 Hz, and 200 ms/6 cycles for 30 Hz). The data in each time window were multiplied with a Hanning taper before Fourier analysis. The power values were obtained for the vertical and horizontal component of the planar gradient and then combined. Finally, the resulting power maps were baseline-corrected using a time window from −0.7 to −0.5 s [relative power change from baseline: (trial − baseline)/baseline].

##### Source reconstruction.

To estimate the underlying brain activity for the alpha band effects found at the sensor level, we performed source reconstruction from-.7 to 2 s. First, a regular grid of 1825 points with 10 mm spacing was created in the Colin27 MRI template (Collins et al., 1998) using Fieldtrip's brain segmentation tools. Then, this set of points was transformed into each participant's space using the individual head shapes derived from the 3D head digitalization. The forward model was solved with a single-shell method and the source reconstruction was performed using the linearly constrained minimum variance (LCMV) beamforming approach implemented in Fieldtrip. We constructed a common filter to ensure reliable comparison between conditions: the spatial filter's coefficients were obtained from the trial-wise covariance matrix from all CR, IR and AR trials and then this filter was multiplied with each condition separately. Before covariance calculation, a principal component analysis (PCA) was conducted, retaining the first 50 components. To maximize the informational content of the signal (Van Veen et al., 1997) while remaining within the functional definition of the alpha band, artifact-free data were initially filtered from 8 to 12 Hz with a Butterworth IIR filter as implemented in Fieldtrip. The final output consisted of a time series estimate per source location, condition and subject.

**Figure 3.**
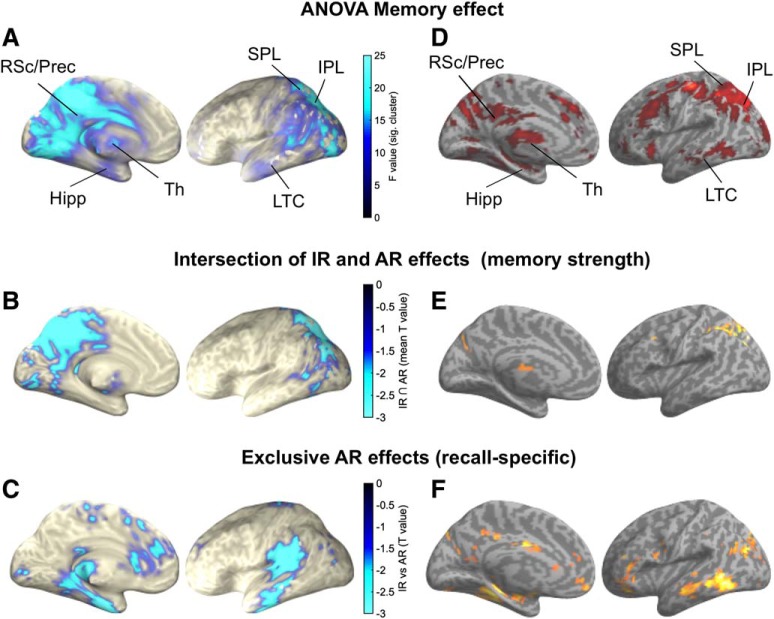
Source reconstruction. ***A***, Significant cluster resulting from the ANOVA in the 10–12 Hz alpha band from 0.7 to 2 s. ***B***, Regions scaling with memory strength (CR < IR < AR), revealed via inclusive masking of condition comparisons (intersection of IR vs CR and AR vs IR) in the 10–12 Hz alpha band and from 1 to 1.5 s. Color bar indicates the mean T values across the IR effect (CR < IR) and the AR effect (IR < AR). ***C***, Exclusive AR effects (recall-specific) map (1 to 1.5 s) indicates areas showing an AR effect (AR > IR, *p* < 0.05, corrected) and no IR effect (IR > CR, *p* < 0.1, uncorrected). Color bar indicates the T value for the AR effect. Labeling of brain regions is based on the Automated Anatomical Labeling (AAL) atlas (Tzourio-Mazoyer et al., 2002). ***D***–***F***, Reanalysis of an fMRI dataset (Staresina et al., 2012) including analogous memory conditions ad contrasts. See text for thresholding settings. For bilateral views and source reconstruction of response-locked data, see Figure 3-1. For pairwise condition comparisons without masking, see Figure 3-2.

Spectral analysis was performed on the reconstructed signal in the same way as in sensor space but restricted to the alpha frequency band (8–12 Hz). To statistically test the sensor-space ANOVA effect (CR, IR, AR) in source space, we averaged baseline-corrected source time series from 10 to 12 Hz from the onset of the effect at 0.7 s to the end of the time period of interest (2 s; Fig. 2*A*) and conducted a repeated-measures ANOVA (Fig. 3*A*). To correct for multiple comparisons across source locations, we used a nonparametric cluster-based permutation test (alpha = 0.05).

## Results

Focusing on correct memory outcomes, our three conditions of interest were: (1) correct rejection of new words (CR), (2) correct identification of old words, without recalling the paired associate (item recognition memory, IR) and (3) correct identification of old words along with correct recall of the paired associate (associative recall, AR). Proportions of trials and reaction times (RTs) are listed in Table 1. The overall rate of HITs (collapsing IR and AR) minus false alarms was 0.59, indicating high levels of recognition memory. The proportion of correct forced choices during the validation task was 0.96 (SEM = 0.01), indicating high levels of paired associate recall after the initial AR response. For analysis of RTs, the median RT for each condition was first derived for each participant. Across participants, RTs differed significantly across our conditions of interest: RTs for Hits were significantly longer than for CR (Wilcoxon *z* = 110, *p* = 0.002), and for IR compared with AR (Wilcoxon *z* = 113, *p* = 0.001). The same statistical pattern was observed when using means and paired-samples *t* tests instead of medians and Wilcoxon tests.

**Table 1.** Retrieval accuracy and reaction times

	Proportion		Reaction times	
	Mean	SEM	Median	IQR
Correct rejections	0.87	0.03	1.32	0.26
Hits	0.72	0.04	1.77	0.53
Associative recall	0.45	0.05	1.52	0.67
Item recognition	0.55	0.05	1.94	0.36

For correct rejections and hits, proportion denotes proportion of all new (112) and old (224) trials, respectively.

### Alpha rhythms track time courses of item recognition and associative recall

Given the RT distribution across trial types (Table 1), we restricted our sensor space analysis to the first 2 s after cue onsets (longest median RT of 1.94 s). To identify - in one step - time points, frequencies and sensors modulated by memory outcome, we first conducted a repeated-measures ANOVA with the factor memory (CR, IR, AR) on time–frequency representations (TFRs, relative power change) across sensors. Results showed a significant effect surviving cluster-based correction for multiple comparisons (Maris and Oostenveld, 2007) (cluster *p* < 0.001). As shown in Figure 2*A*, the effect was centered at left posterior sites, spanning a time window of 0.7–2 s and a frequency range from 8 to 30 Hz, with a distinctive peak from 10 to 12 Hz (alpha frequency range). To maximize sensitivity, subsequent analyses focus on this 10–12 Hz band, but results remain stable when including a wider range of frequencies and sensor selections (Fig. 2-1). When extracting the corresponding power values for the three memory conditions, *post hoc* pairwise tests revealed a stepwise decrease in alpha power from CR to IR (*t*_(14)_ = −4.88, *p* < 0.001, d = 1.26) and from IR to AR (*t*_(14)_ = −4.42, *p* < 0.001, d = 1.14) (Fig. 2*B*). These results extend previous findings of left posterior alpha power distinguishing between correctly recognized old and new items (Hanslmayr et al., 2012), now showing that it further distinguishes between item recognition and associative recall.

10.1523/JNEUROSCI.1982-19.2020.f2-1 Figure 2-1 **Robustness of the sensor-space results to wider range of sensors and frequency bands**. (A) Mean (+/-SEM) power from 0.7-2 s (*left*) and stimulus-locked time courses across participants (*right*) for each memory condition in the 10-12 Hz frequency range (*top*) and 8-20 Hz (*bottom*), collapsed across the 10 sensors showing maximal F values in the main ANOVA (c.f. Figure 2A). (B) Mean (+/-SEM) alpha power from 0.7-2 s (left) and stimulus-locked time courses across participants (*right*) for each memory condition in the 10-12 Hz frequency range, collapsed across the 5 sensors showing maximal F values in the ANOVA. (C) Mean (+/-SEM) alpha power from 0.7-2 s (left) and stimulus-locked time courses across participants (*right*) for each memory condition in the 10-12 Hz frequency range, collapsed across the 20 sensors showing maximal F values in the ANOVA. ***: p < .001 and **: p < .01, paired samples t test. Brown and orange horizontal lines depict the significant clusters for item recognition memory effects (IR vs. CR) and associative recall effects (AR vs. IR), respectively (paired-samples T-tests, all p < .005). Download Figure 2-1, EPS file

10.1523/JNEUROSCI.1982-19.2020.f2-2 Figure 2-2 **Magnetometer sensor and source space ANOVA memory effect**. (A) Sensor space ANOVA results for the comparison of CR, IR and AR TFRs revealed a significant cluster from 0.7-2 s at left posterior sensors with a peak at 10-12 Hz. TFR plot (*left*) depicts the sum of F-values across all significant sensors of the cluster. Topoplot (*right*) shows the sum of F-values across all significant time/frequency bins of the cluster. (B) Source reconstruction results. Significant cluster resulting from the ANOVA in the 10-12 Hz alpha band from 0.7 to 2 s. Download Figure 2-2, EPS file

Do IR and AR effects in the alpha band unfold at different latencies, tracking the delay of recollection relative to familiarity-based recognition (Yonelinas, 2002) or the gradual accumulation of mnemonic evidence (Wagner et al., 2005), respectively? To address this question, we examined the time courses of alpha power at left posterior sensors for CR, IR and AR. As shown in Figure 2*C*, an IR effect emerged at 700 ms post cue onset. Next, with a delay of ∼150 ms, an AR effect emerged as a significant decrease in alpha power for AR relative to IR. To quantify whether alpha power decreases peaked at different latencies for CR, IR and AR, we derived participant-specific time points of maximal alpha power decrease at sensors highligted in Figure 2*A* (dashed red square). Mean peak latencies were 650 ms (SEM = 60 ms) for CR, 786 ms (SEM = 38 ms) for IR and 1043 ms (SEM = 89 ms) for AR. A repeated-measures ANOVA with the factor memory (CR, IR, AR) on these peak latencies confirmed a significant main effect (*F*_(2,28)_ = 9.90, *p* = 0.001, η_p_^2^ = 0.41) with a significant linear term (*F*_(1,14)_ = 13.82, *p* = 0.002, η_p_^2^ = 0.49). *Post hoc* pairwise tests revealed a significant latency difference for AR versus CR (*t*_(14)_ = 3.71, *p* = 0.002, d = 0.96), AR versus IR (*t*_(14)_ = 2.75, *p* = 0.01, d = 0.69) and for IR versus CR (*t*_(14)_ = 2.17, *p* = 0.04, d = 0.56).

Finally, to ensure that our effects do not reflect postretrieval processes (e.g., idling or monitoring, see below), we repeated the time course analysis with response-locked rather than stimulus-locked data, thereby accounting for different response latencies across memory conditions (Table 1). The results confirmed that the differential IR and AR effects unfolded well before the behavioral response: The IR effect emerged ∼950 ms before the response, followed by an AR effect onsetting ∼650 ms before the response (Fig. 2*D*).

### Alpha rhythms track engagement of the core recollection network

As shown in Figure 2*A*, the sensor-level alpha effects were most pronounced over left posterior sites. While this topography is well in line with a host of ERP studies revealing a left posterior recognition memory effect (Sanquist et al., 1980; for a review see Rugg and Curran, 2007), more recent fMRI investigations of recognition memory have consistently revealed a core recollection network, including posterior parietal cortex (PPC) and medial temporal lobe regions. We next projected our data into source space and first focused our source level analysis on the 0.7 to 2 s poststimulus time window and 10–12 Hz frequency band to best capture the memory effects previously found in the sensor-space analysis (Fig. 2). Thresholding the statistical F map from an omnibus ANOVA at *p* < 0.05 (corrected) revealed prominent peaks in medial and lateral PPC (including precuneus, retrosplenial cortex, superior and inferior parietal lobule), lateral temporal cortex (LTC), thalamus, as well as the hippocampus (Fig. 3*A* and Fig. 3-1). Note that a highly similar network emerged when using magnetometer instead of gradiometer data (Fig. 2-2; see also Garcés et al., 2017).

10.1523/JNEUROSCI.1982-19.2020.f3-1 Figure 3-1 **Complete view of the source reconstruction results and source reconstruction of response-locked data**. (A) Significant cluster resulting from the ANOVA in the 10-12 Hz alpha band from 0.7 to 2 s. (B) Regions scaling with memory strength (CR < IR < AR), revealed via inclusive masking of condition comparisons (intersection of IR vs. CR and AR vs. IR) in the 10-12 Hz alpha band and from 1 to 1.5 s. Colorbar indicates the mean T values across the IR effect (CR < IR) and the AR effect (IR < AR). (C) Exclusive AR effects (recall-specific) (1 to 1.5 s) indicates areas showing an AR effect (AR > IR, p < .05, corrected) and no IR effect (IR > CR, p < .1, uncorrected). Colorbar indicates the T value for the AR effect. (D) Significant cluster resulting from the ANOVA on the Response-locked source power in the 10-12 Hz alpha band from -1 to 0 s. Download Figure 3-1, TIF file

10.1523/JNEUROSCI.1982-19.2020.f3-2 Figure 3-2 **Pairwise comparisons** (A) Sensor space T-test results for the comparison of IR and CR TFRs revealed a significant cluster from 0.7-2 s at left posterior and midline sensors from 8 to 30 Hz. TFR plot (A-*left*) depicts the sum of T-values across all significant sensors of the cluster. Topoplot (A-*right*) shows the sum of T-values across all significant time/frequency bins of the cluster. (B) Source reconstruction results. Significant cluster resulting from the T-test in the 8-30 Hz from 0.7 to 2 s. (C) Sensor space T-test results for the comparison of AR and IR TFRs revealed a significant cluster from 0.7-2 s at left posterior sensors from 8 to 15 Hz. TFR plot (C-left) depicts the sum of T-values across all significant sensors of the cluster. Topoplot (C-*right*) shows the sum of T-values across all significant time/frequency bins of the cluster. (D) Source reconstruction results. Significant cluster resulting from the T-test in the 8-15 Hz from 0.7 to 2 s. Download Figure 3-2, EPS file

Within the core recollection network, fMRI studies have consistently revealed functional dissociations, such that PPC regions track memory strength in a monotonic fashion (here: CR < IR < AR), whereas the hippocampus selectively supports recall-based memory (CR = IR < AR) (Hayama et al., 2012; Vilberg and Rugg, 2014). To test whether alpha power source localization is able to track these qualitative differences, we applied inclusive and exclusive masking analyses on the source reconstructed data from 1 to 1.5 s. This time window was chosen because both the IR effect and the AR effect were observable in sensor space (Fig. 2*C*), thus ensuring an unbiased comparison between effects. First, to reveal regions that show a stepwise increase in alpha power desynchronization, we inclusively masked the IR effect (IR > CR) with the AR effect (AR > IR), with both effects thresholded at *p* < 0.05 (corrected). The conjoint effect revealed medial and lateral PPC (Fig. 3*B*). Next, to highlight regions specifically supporting recall in our paradigm, we conducted the contrast of AR > IR (*p* < 0.05, corrected) and excluded regions that would also show an IR effect (IR > CR), liberally thresholded at *p* < 0.1, uncorrected. Note that the more liberal the exclusive mask (here: IR > CR), the more conservative the specificity assessment for the initial contrast (here: AR > IR). This procedure revealed the hippocampus along with lateral temporal cortex (extending into temporoparietal junction) and medial prefrontal cortex (Fig. 3*C*). For more liberal pairwise comparisons without inclusive or exclusive masking, see Figure 3-2. In sum, our MEG source reconstruction analyses revealed a remarkable overlap between the fMRI core recollection network and the regional pattern of alpha power decreases.

Although there are an increasing number of studies reporting reliable MEG source reconstruction of hippocampal signals (for a review see Pu et al., 2018; Ruzich et al., 2019), we took an additional measure to assess our source estimation's reliability. Specifically, we resorted to a previous fMRI dataset that shares some key features with our current study. In that fMRI study, participants encoded word-image pairs during learning (noun-color and noun-scene associations). During subsequent retrieval, they only saw a word (an old target or a novel lure) and indicated with a single button press whether: (1) the given word was new, (2) the given word was old but they could not remember the associated image or (3) the given word was old but and they also remembered the associated image. Analogous to the current paradigm, that study thus yielded three memory conditions of interest: correct rejection of novel words (CR), correct recognition of old words without remembering the associated image (item recognition, IR) and correct recognition of old words along with recalling the associated image (associative recall, AR). For additional details on acquisition and analysis parameters, see Staresina et al. (2012). Importantly, while the original publication focused on a priori MTL regions of interest, we now explored the whole-brain pattern of results to corroborate our MEG alpha power source reconstruction. Of note, during preprocessing, the data were smoothed with a 6 mm FWHM kernel and normalized into MNI space. Functional activation was estimated via a standard GLM procedure, where each event's duration was determined by the trial-specific reaction time (to control for BOLD differences due to RTs alone). For statistics, we used an uncorrected threshold of *p* < 0.001 (minimum 5 contiguous voxels) for the omnibus ANOVA. For the memory strength effect, we inclusively masked the contrast AR > IR with the contrast IR > CR, each thresholded at *p* < 0.001 (uncorrected, minimum 5 contiguous voxels). For the recall specificity analysis, we thresholded the AR > IR contrast at *p* < 0.001 (uncorrected, minimum 5 contiguous voxels) and masked out voxels that would show an IR > CR effect at *p* < 0.1 (uncorrected). As shown in Figure 3, this procedure revealed a remarkable overlap between the two datasets in each of our three memory analyses, emphasizing the merit of source-localizing alpha power effects to unveil specific memory networks.

### Alpha rhythms reveal different temporal profiles within the core recollection network

**Figure 4.**
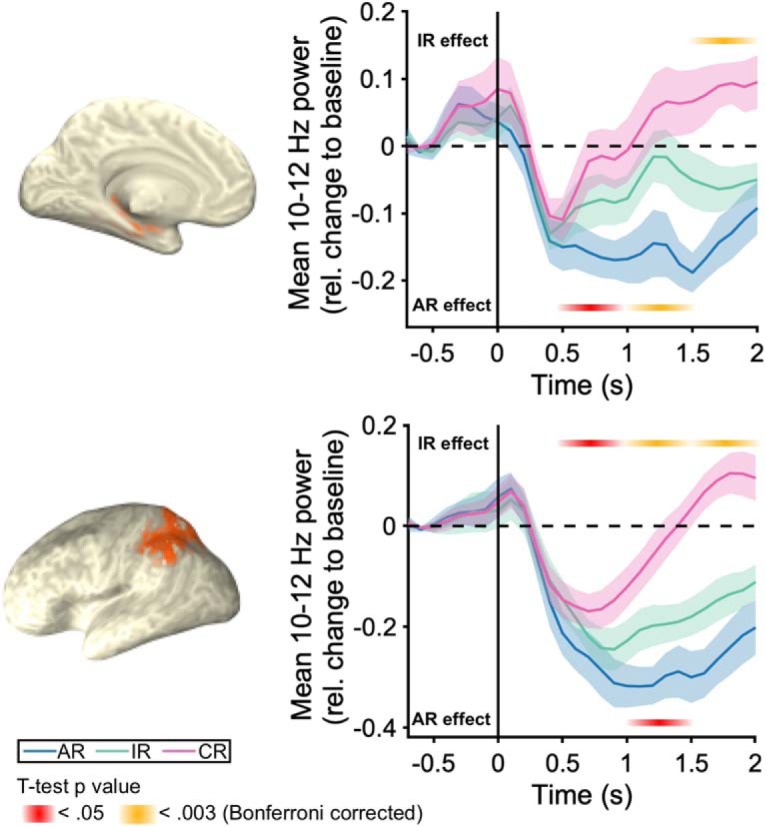
Hippocampus and PPC alpha source power time courses. 10–12 Hz alpha source power for AR, IR and CR. Brain maps depict the regions of interest selected for this analysis based on the AAL atlas (Tzourio-Mazoyer et al., 2002). Top panel depicts alpha power time courses in the hippocampus. Bottom panel includes inferior and superior parietal lobules. Orange and yellow horizontal lines depict significant 500 ms time windows resulting from *post hoc* pairwise tests.

Can the temporal and spatial profiles of alpha power be harnessed to examine the temporal dynamics within the recollection network? Recent fMRI studies have begun to shed some light on the temporal profiles of PPC and hippocampal engagement during retrieval. By varying the interval of maintaining a recalled episodic detail, Vilberg and Rugg (2014) were able to show that hippocampal engagement during successful recall was transient, whereas PPC engagement was sustained and covaried in time with the maintenance interval (see Thakral et al., 2017 for similar results in an episodic future simulation paradigm). While this pattern is consistent with PPC mechanisms being deployed to work with mnemonic content provided by the hippocampus, temporal precedence of a hippocampal relative to a PPC recall effect would provide convergent evidence for this notion. We thus extracted the alpha power time course from PPC and hippocampus (based on bilateral anatomical AAL masks; Tzourio-Mazoyer et al., 2002) to examine a possible temporal dissociation in these regions' memory profiles (Fig. 4). For statistical evaluation, we averaged the baseline-corrected memory conditions across four adjacent 500 ms windows (0–500 ms, 500–1000 ms, 1000–1500 ms and 1500–2000 ms), collapsed them across all virtual voxels in our regions of interest and subjected the resulting data to a Region (hippocampus, PPC) × Condition (CR, IR, AR) × Time repeated-measures ANOVA. Results revealed a significant 3-way interaction (*F*_(6,84)_ = 3.37, *p* = 0.005, η_p_^2^ = 0.194). Follow-up pairwise condition comparisons (paired samples *t* tests) within each region revealed an earlier and stronger AR effect in hippocampus and an earlier and stronger IR effect in PPC. In particular, the comparison of AR versus IR was significant in hippocampus in the 500–1000 ms time window (*t*_(14)_ = −2.9, *p* = 0.01, d = 0.49) and in the 1000–1500 ms time window (*t*_(14)_ = −3.61, *p* = 0.0028, d = 0.93), but only significant in PPC in the 1000–1500 ms time window (*t*_(14)_ = −3.40, *p* = 0.004, d = 0.87). When applying a stringent Bonferroni correction for our total of 16 comparisons (*p* < 0.0031), only the hippocampal AR effect from 1000 to 1500 ms survived. Conversely, the comparison of IR versus CR was significant in PPC in all time windows from 500 to 2000 ms (all *t*_(14)_ ≥ −0.16, *p* < 0.03, d > 0.64), but only significant in hippocampus in the late 1500–2000 ms time window (*t*_(14)_ = −4.59, *p* = 0.0004, d = 1.18). The PPC IR effect survived Bonferroni correction from 1000 to 2000 ms and the hippocampal IR effect survived Bonferroni correction from 1500 to 2000 ms. These patterns point to a recall-specific signal in the hippocampus, which is followed by PPC recruitment, with the latter possibly reflecting the additional amount of memory strength/mnemonic detail (Wagner et al., 2005; Rugg and Vilberg, 2013) and/or attention to memory (Ciaramelli et al., 2010). Conversely, the delayed IR effect in hippocampus strongly resembles a previous iEEG report that showed the same effect sequence in hippocampus (i.e., an AR effect preceding an IR effect; Staresina et al., 2012) and might reflect hippocampal encoding operations deployed for novel stimuli (see also Okado and Stark, 2005).

## Discussion

Our results show that alpha power desynchronization in MEG unifies previous studies of the temporal (EEG) or spatial (fMRI) profiles of memory retrieval. Despite the long history of M/EEG studies on recognition memory (Sanquist et al., 1980; for reviews, see Mecklinger, 2000; Rugg and Curran, 2007), only a few have examined oscillatory patterns related to different memory outcomes (Burgess and Gruzelier, 2000; Khader and Rösler, 2011; Michelmann et al., 2016; Waldhauser et al., 2016; Vogelsang et al., 2018), though without explicitly distinguishing associative recall from item recognition. Our paradigm (Fig. 1) allowed us to directly probe the oscillatory patterns that support these different memory signals. As shown in Figure 2, the results revealed that left posterior alpha desynchronization not only tracked simple old/new recognition memory (IR vs CR), but further distinguished between old/new recognition and associative recall (AR vs IR). Indeed, time course analyses (Fig. 2*C*,*D*) confirmed the temporal offset between an earlier IR effect (starting at ∼700 ms after cue onset) followed by a later AR effect (starting at ∼900 ms after cue onset) (Yonelinas, 2002; Rugg and Yonelinas, 2003). We note that the onset latency of the IR effect is markedly later than the FN400 component (negative signal deflection over frontal sites around 400 ms) traditionally linked to familiarity-based recognition (Düzel et al., 1997; Curran, 2000; Johansson and Mecklinger, 2003; Rugg and Curran, 2007). We thus suggest that the stepwise change in alpha power at left posterior sites, including a stepwise delay in peak latencies (CR < IR < AR), reflects the gradual accumulation of memory strength/mnemonic evidence (Wagner et al., 2005). In any case, considering the potential link between amplitude fluctuations in the alpha band and sustained ERP deflections (Mazaheri and Jensen, 2008), our data raise the speculative possibility that at least some of the classic sustained/late ERP recognition effects reflect condition-specific differences in alpha power.

In a separate line of research, fMRI studies on recognition memory have consistently shown engagement of a particular set of brain regions in recall-based memory, including lateral/medial parietal and temporal regions. The robustness of these regions' engagement across numerous paradigms has given rise to the notion that they represent a core recollection network (Rugg and Vilberg, 2013). However, given the relatively poor temporal resolution of fMRI, it has been challenging to pinpoint the exact cognitive (sub)processes that these regions support during recognition memory. Accordingly, while some accounts posit that this network represents the information retrieved from long-term memory (Johnson et al., 2013; Rugg and Vilberg, 2013; Vilberg and Rugg, 2014; Thakral et al., 2017), others highlight—particularly regarding parietal contributions—various types of preretrieval (Cabeza, 2008), periretrieval (Wagner et al., 2005; Haramati et al., 2008; Shimamura, 2011) or other postretrieval (Ciaramelli et al., 2010) operations (for reviews see Levy, 2012; Sestieri et al., 2017). Projecting our sensor data into source space, we found a strong overlap of our alpha power memory effects with the core recollection network (Fig. 3). The pairwise comparisons showed that both IR and AR effects map onto bilateral (superior/inferior parietal lobule) and medial (precuneus/retrosplenial cortex; see also Bergström et al., 2013) parietal cortex. Conversely, hippocampus and medial prefrontal cortex showed specific engagement for AR (Fig. 3*C*). The topographical correspondence of our alpha power decreases with BOLD increases commonly found in fMRI recognition memory studies adds to a number of EEG-fMRI studies showing a tight coupling of these two measures (Laufs et al., 2006). To further corroborate the similarity of our MEG results with fMRI findings, we reanalyzed an fMRI study in which comparable memory processes were examined (i.e., CR, IR and AR; Staresina et al., 2012). As shown in Figure 3, there was a remarkable overlap in topographies of different memory effects between the source-localized MEG alpha power data (Fig. 3, left) and the analogous fMRI BOLD contrasts (Fig. 3, right). This suggests that alpha power can, at least to a certain extent, be used as a time-resolved proxy for BOLD activation in memory paradigms.

The yoking of alpha desynchronization effects with the fMRI recollection network opens insights into this network's temporal profile and informs theories on hippocampal and PPC contributions to memory retrieval. First, taking sensor space (Fig. 2*C*) and source space temporal dynamics (Fig. 4) together, the memory effects clearly emerged after cue onset but well before the mnemonic decision (median RT = 1.77 s), pointing to periretrieval engagement of the recollection network rather than prestimulus preparatory or postretrieval monitoring/decision making functions. Moreover, across hippocampus and PPC, the source power time courses (Fig. 4) suggest that recall success is initiated by the hippocampus and subsequently PPC might govern the ensuing accumulation of mnemonic evidence and/or provide an 'episodic buffer' (Baddeley, 2000; Shimamura, 2011; Hayama et al., 2012; Rugg and Vilberg, 2013).

The link between parietal alpha power decreases and the accumulation of mnemonic evidence also aligns with a recent account of alpha oscillations (Hanslmayr et al., 2012, 2016,). That is, although the exact functional significance of alpha oscillations is still debated [e.g., “idling” (Pfurtscheller and Lopes da Silva, 1999) vs “active inhibition” (Klimesch, 1996; Jensen and Mazaheri, 2010)], modeling (Parish et al., 2018) and empirical data (Griffiths et al., 2019) suggest that cortical low-frequency (alpha/beta, i.e., ∼8–30 Hz) desynchronization reflects the amount of information and the fidelity of memory reinstatement in a given region (Hanslmayr et al., 2012, 2016; Griffiths et al., 2019). In the hippocampus, the alpha power decrease for AR versus IR may again reflect an increase in memory reinstatement. The MEG alpha power decrease in the hippocampus observed here is highly similar to that shown in a recent iEEG study using direct hippocampal recordings (Staresina et al., 2016), both in the frequency range and effect latency. In that study, the alpha power decrease for associative versus nonassociative retrieval (similar to AR vs IR here) coincided with event-specific memory reinstatement and was preceded by a gamma power (∼50–90 Hz) increase at 500 ms. One plausible scenario might thus be that the gamma power increase at 500 ms reflects hippocampal pattern completion processes, with the ensuing alpha power decrease reflecting an increase in reinstated mnemonic content emerging from this process (Staresina and Wimber, 2019). Of course caution is warranted when interpreting MEG effects in deep anatomical sources such as the hippocampus, but in addition to the convergence of results with direct iEEG recordings and fMRI data, our findings add to a growing body of evidence of discernible MEG effects in the hippocampus (for a review see Pu et al., 2018; Ruzich et al., 2019).

Finally, while our effects were most prominent in the alpha frequency band (Fig. 2*A*), it is important to note that other low-frequency bands, particularly theta (4–8 Hz), have also been linked to memory processes. For instance, Osipova et al. (2006) found theta increases for HITs relative to CRs in an image recognition paradigm. Interestingly, though, this effect was localized to occipital cortex and already started 300 ms post cue onset. Theta power increases have also been linked to hippocampal retrieval process in iEEG recordings (Burke et al., 2014), though that study used a free recall paradigm rather than a recognition memory/cued recall paradigm. Another recent study combined MEG recordings with continuous theta burst stimulation (cTBS) during an autobiographical memory task (Hebscher et al., 2019) and found increased theta power and theta-gamma coupling in the core recollection network. Together, this raises the possibility that different functional networks, recruited by different memory demands, are grouped by different frequency bands, and an important challenge for future studies will thus be to delineate the roles of theta power increases versus alpha power decreases in service of episodic retrieval (Hanslmayr et al., 2016).

To conclude, our understanding of recognition memory has thus far relied upon separate lines of research capitalizing on either temporal or spatial signal properties. Our study suggests that alpha rhythms represent a single oscillatory measure tracking when and where item and associative memory unfolds in time and space, unveiling differential engagement of the hippocampus and parietal cortex at different stages of episodic retrieval.

## Data Availability

Raw MEG data and analysis scripts can be downloaded from https://www.mrc-cbu.cam.ac.uk/publications/opendata/.
