# Supplementary figures and images for: Alpha Rhythms Reveal When and Where Item and Associative Memories Are Retrieved
Source: J Neurosci. 2020 Mar 18;40(12):2510–8. doi: 10.1523/JNEUROSCI.1982-19.2020 (PMC7083536; doi:10.1523/JNEUROSCI.1982-19.2020)

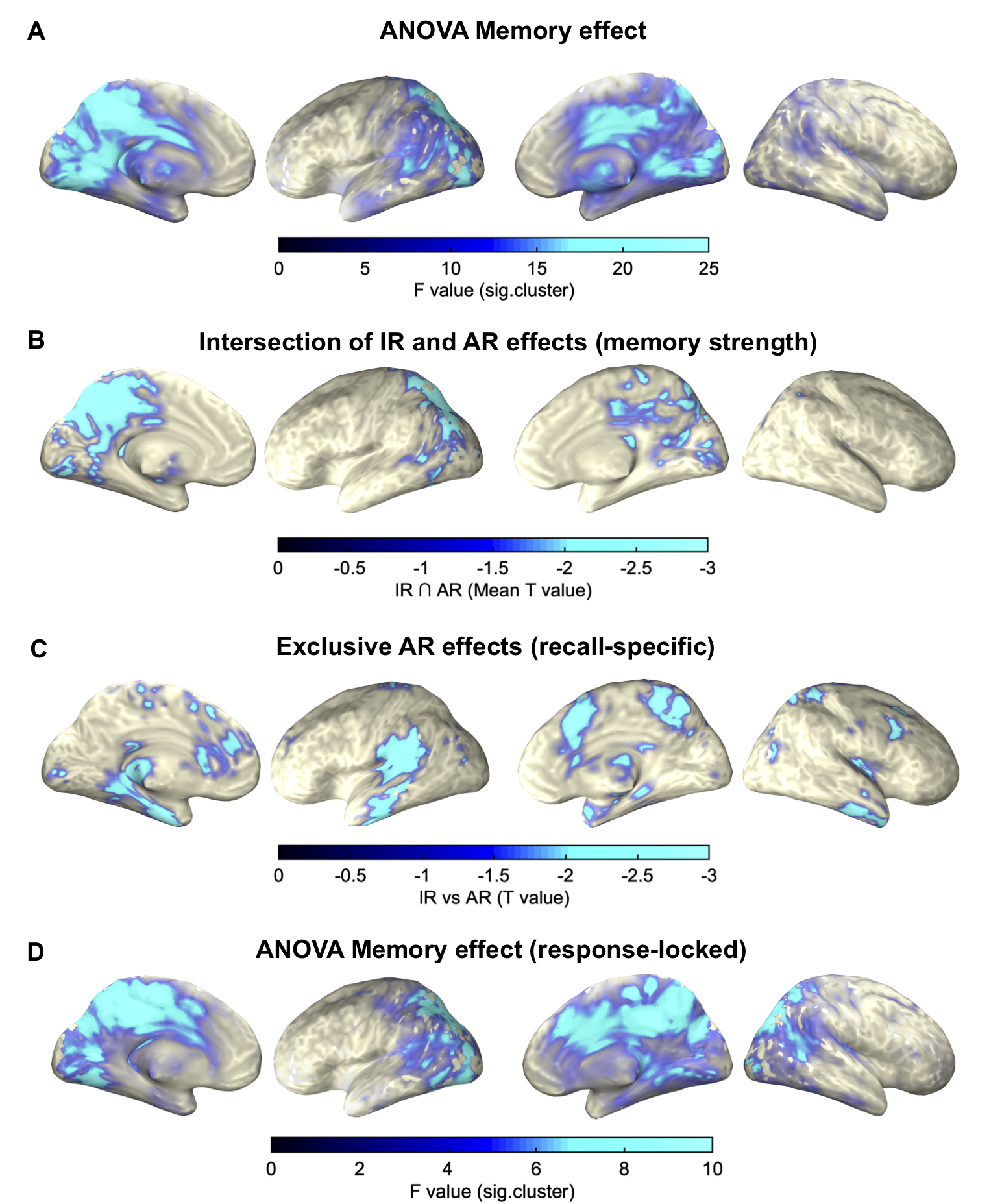

Supplement: Figure 3-1 [file sup_ns-JN-RM-1982-19-s04.tif]
